# Unraveling the Overlapping Spectrum of Hereditary Neuropathies: Clinical and Genetic Insights From the UAE

**DOI:** 10.7759/cureus.93582

**Published:** 2025-09-30

**Authors:** Adil Jumani, Ghada Rashwan, Hadiza Ibrahim, Hesham Eissa, Mohamed E Abouelnaga, Amani Alzaabi, Mahfoud Elbashari

**Affiliations:** 1 Internal Medicine, Zayed Military Hospital, Abu Dhabi, ARE; 2 Neurology - Internal Medicine, Zayed Military Hospital, Abu Dhabi, ARE

**Keywords:** charcot-marie-tooth disease, hereditary neuropathy with liability to pressure palsy (hnpp)), hereditary sensory and autonomic neuropathies (hsan), neuromuscular diseases, peripheral neuropathy

## Abstract

Hereditary sensory and motor neuropathies (HSMNs) remain underreported in the United Arab Emirates (UAE), where overlapping clinical features often delay diagnosis. These case series describe the clinical and genetic diversity of HSMNs through the evaluation of five Emirati male patients, aged 18-47 years diagnosed with Charcot-Marie-Tooth disease (CMT) and Hereditary Neuropathy with Liability to Pressure Palsies (HNPP), focusing on phenotypic variability and diagnostic challenges. Patients presented with a range of symptoms, from episodic focal neuropathies to progressive distal weakness and motor impairment. Clinical assessment, electrophysiological studies and genetic testing revealed a number of underlying mutations, including PMP22 duplications (CMT1A), PMP22 deletions (HNPP), GJB1 mutation (CMTX), and SH3TC2 mutation (CMT4C). The CMT4C case exhibited early-onset scoliosis and severe neuropathy, while others exhibited milder or episodic symptoms. One HNPP patient with a PMP22 deletion exhibited progressive deficits resembling CMT, emphasizing the phenotypic overlap between HNPP and CMT. Electrophysiological studies showed demyelinating polyneuropathy with varying degrees of axonal involvement. These findings highlight the challenges posed by HSMNs and the importance of integrating clinical assessment, electrophysiological evaluation, and comprehensive genetic testing for accurate diagnosis and management. This multidisciplinary approach is critical for distinguishing between CMT and HNPP, offering clarity in cases with atypical or overlapping phenotypes and supporting the need for personalized care.

## Introduction

Hereditary sensory and motor neuropathies (HSMNs) are a group of inherited disorders characterized by progressive dysfunction of peripheral nerves, leading to motor and sensory deficits. Among these, Charcot-Marie-Tooth disease (CMT) and Hereditary Neuropathy with Liability to Pressure Palsies (HNPP) are prominent subtypes [1]. CMT typically presents as chronic, progressive neuropathy with distal muscle weakness, atrophy, and sensory loss, often with a gradual onset [2]. In contrast, HNPP is marked by episodic, painless, recurrent focal neuropathies triggered by minor compression or trauma to nerves, resulting in transient numbness, tingling, and muscle weakness [3]. Both conditions share a genetic basis involving the PMP22 gene, which is crucial for myelin sheath formation in peripheral nerves. Duplication causes Charcot-Marie-Tooth disease type 1A (CMT1A) by overexpressing PMP22, resulting in abnormal myelin formation, chronic demyelination, and progressive distal weakness. In contrast, deletion leads to HNPP through PMP22 haploinsufficiency, producing unstable, thin myelin that's vulnerable to mechanical stress, causing transient, recurrent mononeuropathies. Thus, the dosage sensitivity of PMP22 disrupts myelin homeostasis in opposite ways, leading to different clinical phenotypes [4]. These case series explore the variability in clinical presentations and disease progression of CMT and HNPP, emphasizing the diagnostic challenges and the crucial role of genetic testing in confirming the diagnosis.

## Case presentation

We reviewed a total of five patients for our series after obtaining ethical approval from the ethical committee at Zayed Military Hospital. Their demographic details, clinical presentations, examination findings, electrophysiological results, and genetic diagnoses are summarized in Table 1.

**Table 1:** Clinical, electrophysiological, and genetic characteristics of five Emirati male patients with hereditary sensory and motor neuropathies (HSMNs), including Charcot-Marie-Tooth disease (CMT) and hereditary neuropathy with liability to pressure palsies (HNPP).

Case No.	Age (years)	Clinical Presentation	Examination findings	Family history	Nerve conduction study	Gene identified	Diagnosis
1	19	Bilateral hand tremors, difficulty walking and numbness in the feet.	Mild hand muscle wasting, tremors, bilateral foot drop, depressed reflexes	Yes	Primary demyelination with secondary axonal loss in both upper and lower limbs.	Variant in GJB1 gene	Charcot-Marie-Tooth (CMT) type X1
2	18	Numbness of medial aspects of both arms, injury-related numbness over the right leg.	Decreased sensation in both arms medially and the right leg laterally	No	Demyelinating and axonal sensorimotor polyneuropathy mainly in both legs.	PMP22 gene deletion	Hereditary neuropathy with liability to pressure palsies (HNPP)
3	19	Bilateral lower limb weakness.	Power 4/5 LL, reflexes present with reinforcement, glove-stocking sensory loss.	No	Distal demyelinating sensorimotor polyneuropathy	PMP22 gene deletion	Hereditary neuropathy with liability to pressure palsies (HNPP)
4	47	Occasional numbness in the upper extremities followed by weakness of the left side of the body.	Lost dorsi and plantar flexion of the feet. Absent bilateral knee and ankle reflexes. Foot drop bilaterally.	Yes	Sensori-motor axonal polyneuropathy.	SH3TC2 gene	Charcot-Marie-Tooth (CMT) type 4C
5	30	Bilateral hand tremors and numbness.	Moderate distal hand tremors, otherwise unremarkable.	No	Mild demyelinating neuropathy in both upper limbs.	PMP22 gene duplication	Charcot-Marie-Tooth (CMT) type 1A

Case description

Case 1

**Figure 1:**
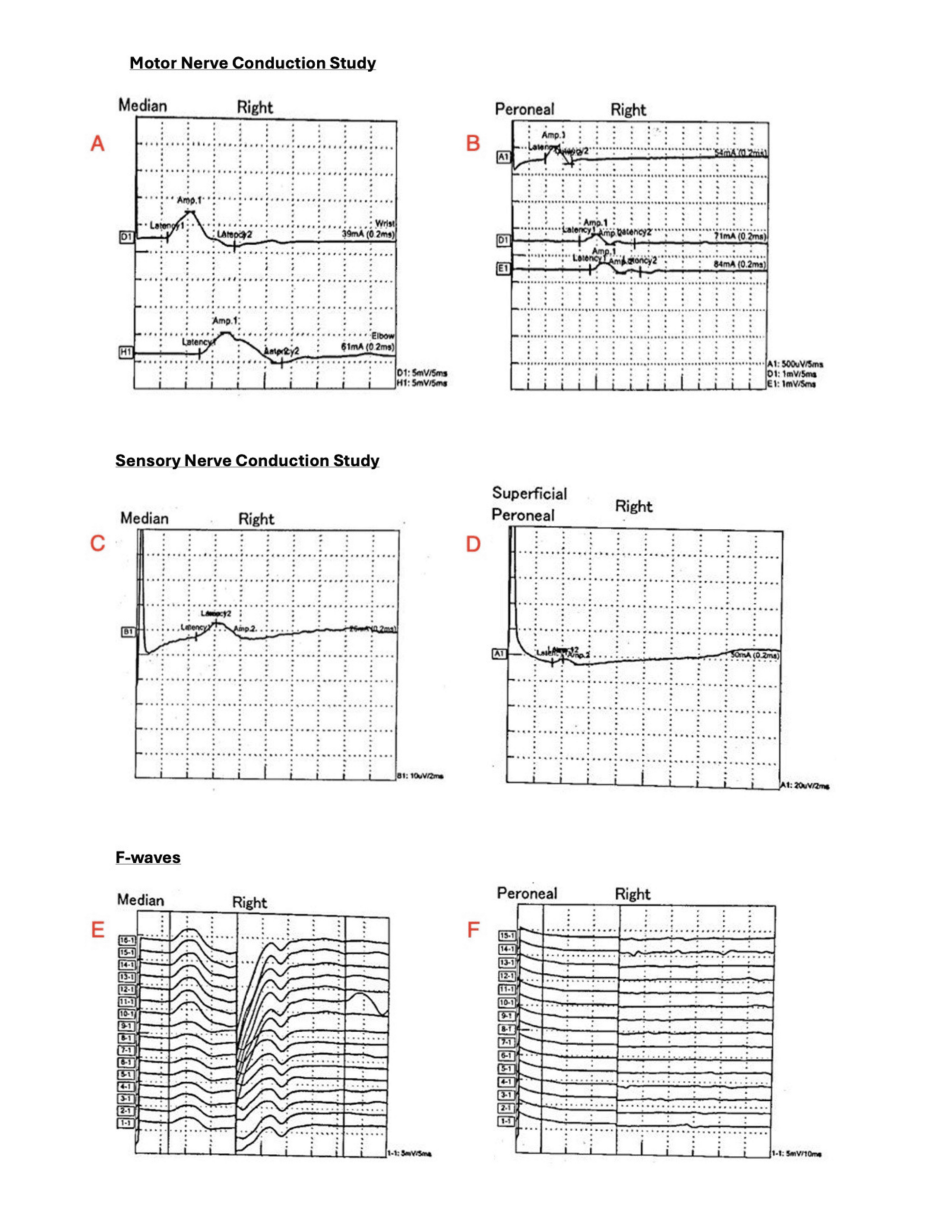
Nerve conduction study findings in Charcot-Marie-Tooth (CMT). (A) Right median motor nerve conduction showing prolonged distal latency and reduced compound muscle action potential (CMAP) amplitude. (B) Right peroneal motor nerve conduction demonstrating slowed conduction velocity and reduced CMAP amplitude. (C) Right median sensory nerve conduction with prolonged latency and low sensory nerve action potential (SNAP) amplitude. (D) Right superficial peroneal sensory nerve conduction showing delayed latency. (E) Right median F-waves demonstrating prolonged latencies. (F) Right peroneal F-waves with absent late responses. Abbreviations: CMAP = compound muscle action potential; SNAP = sensory nerve action potential. Units: ms = milliseconds; mA = milliampere; µV = microvolt.

A 19-year-old male presented to the neurology clinic with complaints of tremor of the hands and difficulty walking since the age of 11. He described his difficulty walking as his knees suddenly giving off with twisting of his feet. He also complained of numbness in his feet. He reported symptoms of foot weakness in his brother and sister, and that they usually walk on their toes. His maternal aunt also had similar complaints. On examination, he had mild wasting of the small muscles of the hand and tremors in the outstretched hand. He had bilateral foot drop and depressed reflexes in the upper and lower limbs. His nerve conduction study showed features suggestive of demyelinating axonal neuropathy in both upper and lower limbs. He underwent genetic testing to identify abnormalities in the PMP22 gene, which was reported normal. Given his highly suggestive clinical picture along with nerve conduction study findings, a next-generation sequencing analysis of all known genes associated with CMT was performed, which showed a hemizygous variant in the GJB1 gene. He was subsequently diagnosed with Charcot-Marie-Tooth disease type 1 (Figure 1).

Case 2

An 18-year-old male presented to the neurology clinic with complaints of numbness in the arms and right leg. He stated that he started to have numbness in his left arm after a fall injury sustained eight years prior to presentation while playing football. He underwent surgical repair of a partial cut of the left radial nerve at the spinal groove, which significantly improved his symptoms; however, he had residual numbness over the same arm. On examination, he was found to have decreased sensation over the medial aspect of both arms and decreased sensation over the lateral aspect of the right leg. He was not aware of similar complaints in any other family member.

**Figure 2:**
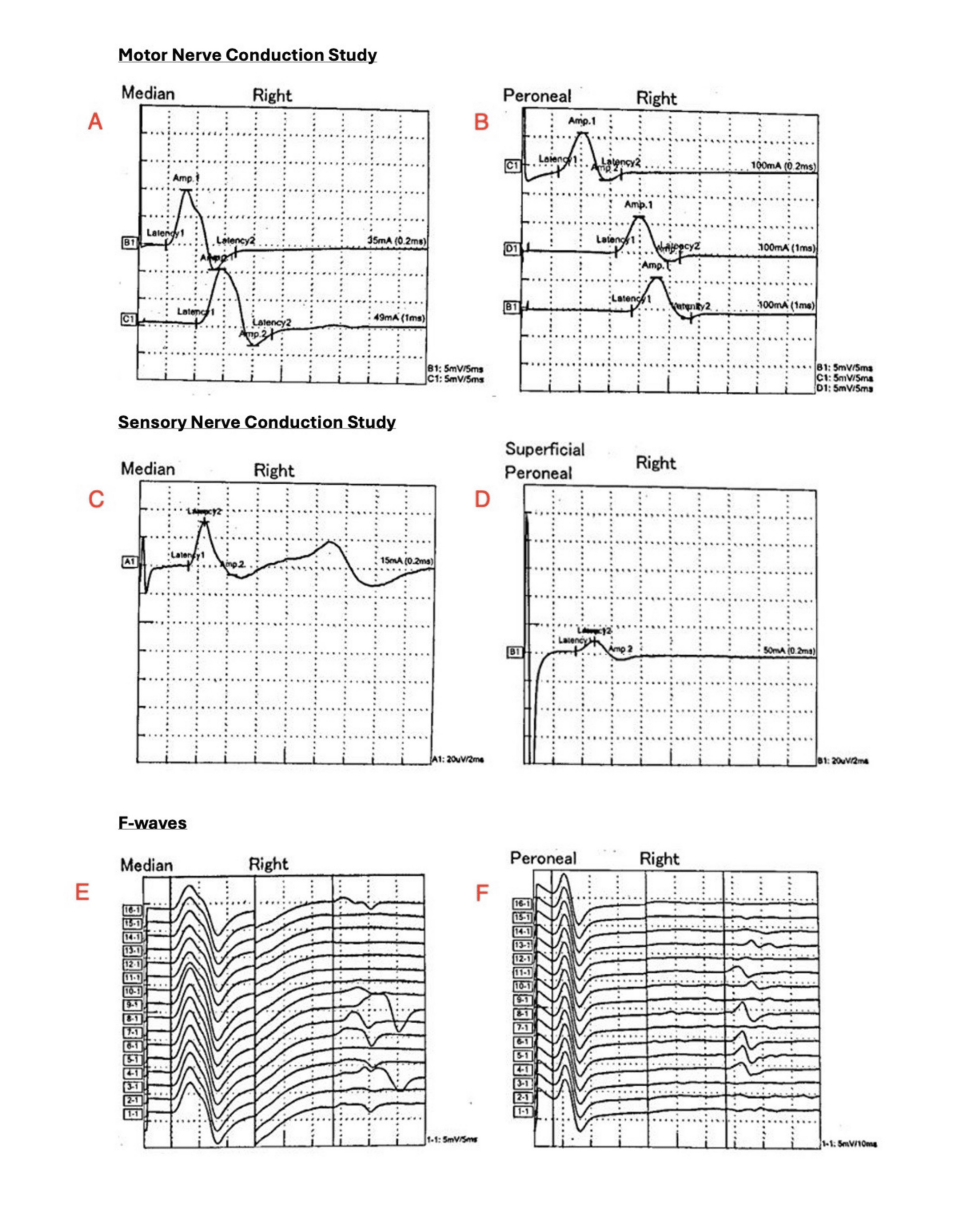
Nerve conduction study findings in hereditary neuropathy with liability to pressure palsies (HNPP). (A) Right median motor nerve conduction showing prolonged distal latency with preserved CMAP amplitude. (B) Right peroneal motor nerve conduction demonstrating multifocal slowing at entrapment sites. (C) Right median sensory nerve conduction with delayed latency and reduced SNAP amplitude. (D) Right superficial peroneal sensory nerve conduction showing markedly delayed response. (E) Right median F-waves demonstrating prolonged latencies. (F) Right peroneal F-waves with dispersed late responses. Abbreviations: CMAP = compound muscle action potential; SNAP = sensory nerve action potential. Units: ms = milliseconds; mA = milliampere; µV = microvolt.

A nerve conduction study was done, which showed mild to moderate predominantly demyelinating and axonal sensorimotor polyneuropathy involving both legs, with left leg involvement more than the right. Given the clinical picture and nerve conduction study findings, genetic testing was done, which was positive for PMP22 gene deletion in keeping with the diagnosis of hereditary neuropathy with liability to pressure palsies (Figure 2).

Case 3

**Figure 3:**
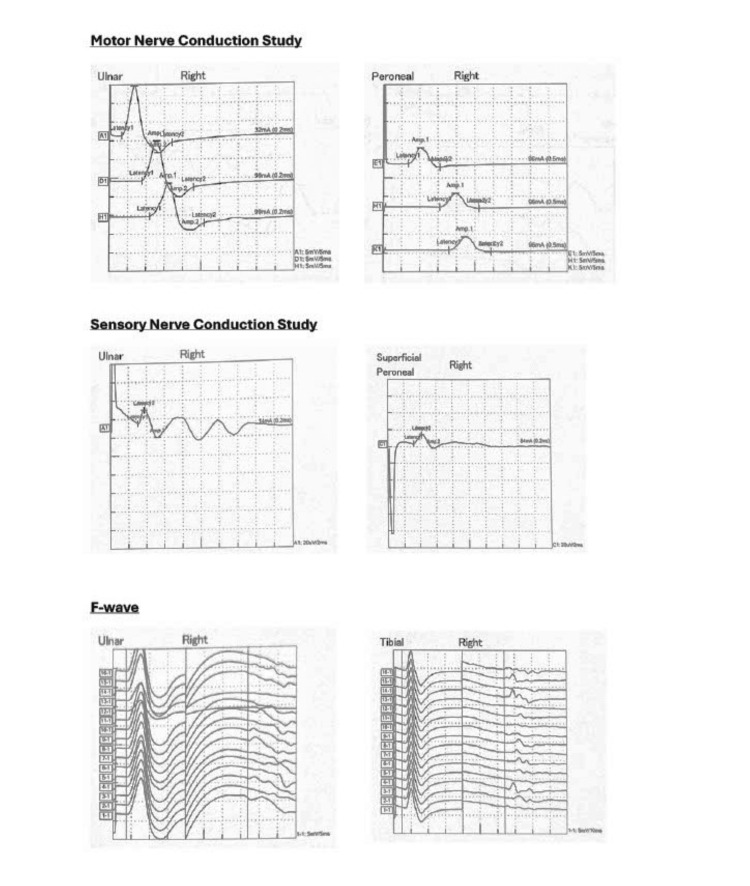
Nerve conduction study showing mild to moderate predominantly distal demyelinating sensorimotor polyneuropathy.

A 19-year-old male presented to the neurology clinic with a history of weakness and numbness in the lower limbs, causing difficulty in walking. Prior to this, he had presented to the emergency department with similar complaints twice. The first episode was acute; he had gone to sleep, and when he woke up in the middle of the night, he was unable to walk. He was admitted and investigated with lumbar puncture and MRI imaging of the brain, cervical spine, and lumbosacral spine, all of which were unremarkable. His examination was positive for a power of 4/5 in the lower limbs. Reflexes were present only with reinforcement. His plantars were downgoing bilaterally. He had loss of sensation in a glove-stocking distribution in the lower limbs. His vasculitis screen and anti-MOG antibodies were negative. He had a nerve conduction study done, which showed mild to moderate predominantly distal demyelinating sensorimotor polyneuropathy. Based on this and persistence of symptoms with otherwise negative laboratory and radiological workup, he had a genetic test done, which showed PMP22 gene deletion, consistent with the diagnosis of hereditary neuropathy with liability to pressure palsies (Figure 3).

Case 4

**Figure 4:**
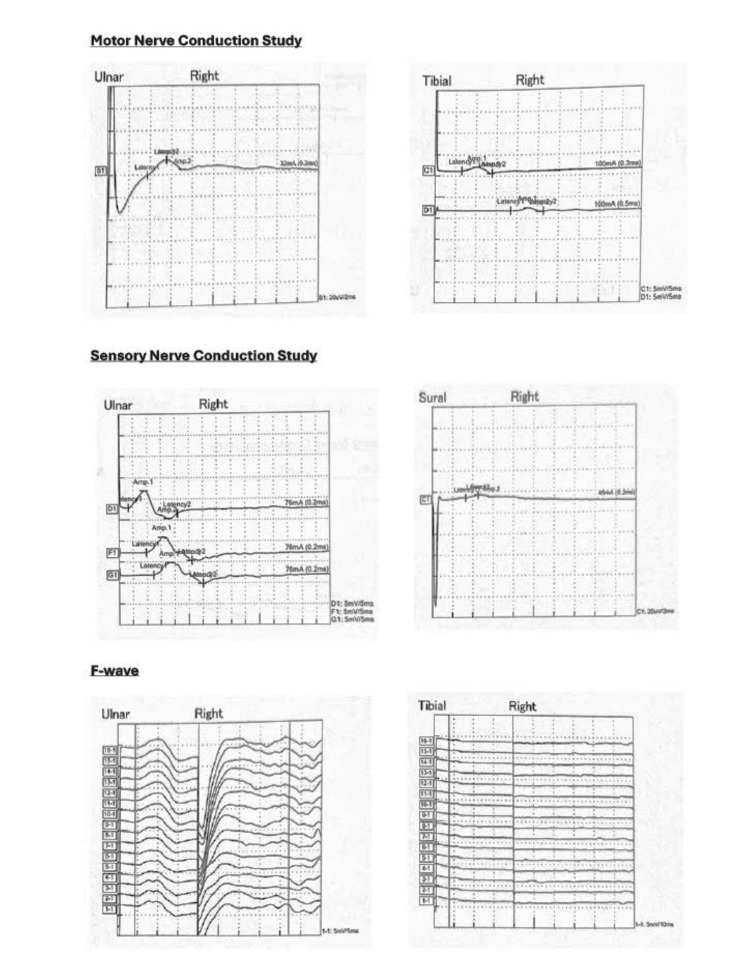
Nerve conduction study showing moderately severe sensorimotor polyneuropathy with axonal degenerative changes predominantly in the lower limbs.

A 47-year-old male with a medical history of diabetes, dyslipidemia and obesity presented to the emergency department with acute left-sided body weakness and was subsequently diagnosed with bilateral stroke. He was fully evaluated by a neurologist at the time due to his primary diagnosis, and he also disclosed complaints of numbness intermittently affecting his upper extremities that started from childhood. He states that he started wearing closed-off shoes for more support, he is unable to climb stairs without assistance and has had recurrent minor falls. He reported his leg weakness was progressive, and as a result, he switched from a field job to an administrative job. He also reports similar complaints in his younger brother, who became wheelchair bound at the age of 25 years. On examination, he was found to have foot drop with weakness in toe adduction and abduction, absent bilateral knee and ankle reflexes. This triggered a workup for polyneuropathy. Nerve conduction studies were done and showed motor-sensory axonal polyneuropathy concerning for hereditary neuropathy. He underwent genetic testing, which revealed a pathogenic variant in the SH3TC2 gene consistent with the diagnosis of CMT type 4C (Figure 4).

Case 5

**Figure 5:**
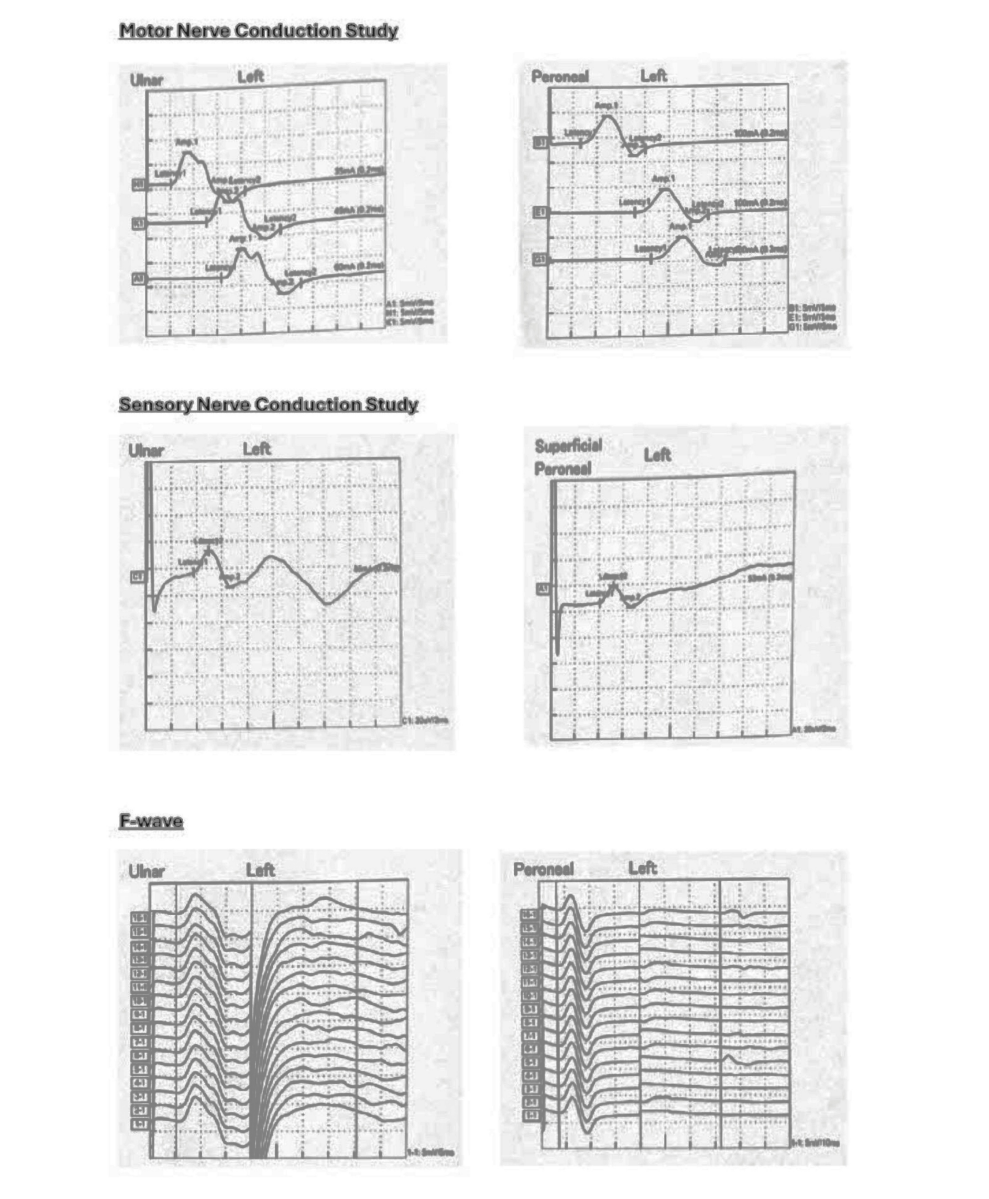
Nerve conduction study showing predominantly demyelinating diffuse sensorimotor polyneuropathy.

A 30-year-old male presented to the neurology clinic with complaints of tremors in both hands for the last two years. He stated that tremors were worse with stress, caffeine, and exercise. He also had occasional spillage of liquids due to difficulty holding objects. He noticed a new onset of numbness in his hands for the last two months, which was worse at night. He was diagnosed with benign essential tremor and was started on propranolol 10 mg thrice daily. On examination, he had moderate distal tremors of both hands with an otherwise unremarkable exam. He had laboratory tests done, which showed normal thyroid function. He was scheduled for a nerve conduction study, which showed bilateral mild demyelinating neuropathy in the upper limbs and significantly decreased compound motor action potential (CMAP) of the left peroneal nerve when compared to the right. Due to the presence of polyneuropathy, the patient underwent genetic testing for the possibility of CMT. Genetic testing showed duplication of the PMP22 gene consistent with CMT. The patient was started on pregabalin 75 mg at bedtime and was referred to physical and occupational therapy. Pregabalin dose was gradually increased to 300 mg daily, after which the patient reported significant improvement of symptoms (Figure 5).

## Discussion

This case series of five male patients highlights the broad clinical heterogeneity of HSMNs. All patients manifested a chronic length-dependent sensorimotor polyneuropathy - characterized by distal muscle weakness/atrophy, depressed reflexes, and mild distal sensory loss - consistent with the general phenotype of CMT neuropathy [5]. However, the severity and specific features varied widely. One patient had an early childhood-onset, severe demyelinating neuropathy with pronounced distal weakness and skeletal deformities (including foot deformities and progressive scoliosis), whereas another patient experienced only episodic, transient neuropathic deficits with near-complete recovery between episodes. The remaining patients fell between these extremes, with differing ages of onset (ranging from the first to fourth decade) and degrees of motor versus sensory involvement. This spectrum exemplifies the well-known phenotypic variability in HSMNs [6].

The genetic basis of HSMNs contributes to their clinical heterogeneity. While CMT1A and HNPP are primarily linked to PMP22 gene duplication and deletion, respectively, other genetic mutations, such as those in the GJB1 and SH3TC2 genes, significantly alter the disease phenotype, as seen in our cases. For example, the CMT1A patient (with a PMP22 duplication) developed a classic CMT1 phenotype: onset in the second decade with slowly progressive, symmetric distal weakness and atrophy (legs more affected than arms), length-dependent sensory loss, and difficulty running in adolescence, which is consistent with the published cohorts where patients with CMT1A typically present by the first two decades and progresses gradually, with most patients remaining ambulatory for life and normal life expectancy [7].

The X-linked CMT patient in our series (with a GJB1 mutation, CMT1X) demonstrated a phenotype similar to males with CMT1 reported in the literature, with some nuances due to the X-linked genetics. He developed symptoms in late adolescence, including difficulty with foot dorsiflexion and mild hand weakness, and had a steadily progressive course of distal weakness and sensory loss over the next decade. Our patient’s need for orthotic support for foot drop by his 20s is consistent with the moderate disability typical of CMT1X in males [8]. This emphasizes the importance of broad genetic screening when PMP22 abnormalities are not detected.

The most severe neuropathy in our series was seen in the CMT4C patient (SH3TC2 mutation, which highlights the autosomal recessive nature of certain subtypes with scoliosis and early severe disability). He had the onset of walking difficulty in early childhood (<5 years old), developed marked distal weakness in both legs and hands during school-age years, and by age 18 had lost the ability to run and had significant orthopaedic complications. Most patients with CMT4C develop thoracic scoliosis in childhood and have moderate-to-severe peripheral neuropathy, as seen in our patient [9].

​In our series, both patients were diagnosed with HNPP based on clinical features and confirmed PMP22 deletions. Both showed demyelinating sensorimotor polyneuropathy on nerve conduction studies, with multifocal slowing at typical entrapment sites. However, one patient exhibited more progressive symptoms with persistent distal sensory loss and motor deficits, a departure from the classic transient pattern. This divergence is increasingly recognized in the literature. Li et al. described patients with confirmed PMP22 deletions, where some individuals showed progressive deficits rather than the classical transient episodes, closely resembling CMT ​[10]. This overlap between CMT and HNPP is well recognized. Some individuals with PMP22 deletions can exhibit features resembling CMT rather than the typical episodic neuropathies of HNPP, suggesting a continuum of phenotypic expression.

Electrophysiological studies remain a key diagnostic tool; however, their interpretation requires correlation with clinical findings and genetic results. The presence of demyelinating neuropathy with variable axonal involvement, as seen in our cases, aligns with findings in previous studies that emphasize the role of electrophysiology in differentiating between HNPP and CMT [11]. Furthermore, a systematic review by Murphy et al. highlighted the challenges in distinguishing CMT from HNPP based solely on electrophysiologic criteria, stressing the necessity of a comprehensive diagnostic approach, including genetic testing [12].

## Conclusions

This paper highlights the clinical variability between CMT and HNPP. Despite sharing an underlying peripheral demyelinating pathology, their phenotypic presentations span a broad clinical spectrum, often complicating the diagnostic process. Our findings reinforce the importance of a comprehensive approach that combines clinical evaluation, electrophysiological studies, and genetic testing to establish an accurate diagnosis. A multidisciplinary diagnostic approach is essential for accurate classification, management and genetic counseling in such patients.
